# Analysis of the Impact of a Multi-Strain Probiotic on Body Composition and Cardiorespiratory Fitness in Long-Distance Runners

**DOI:** 10.3390/nu12123758

**Published:** 2020-12-07

**Authors:** Joanna Smarkusz-Zarzecka, Lucyna Ostrowska, Joanna Leszczyńska, Karolina Orywal, Urszula Cwalina, Damian Pogodziński

**Affiliations:** 1 Department of Dietetics and Clinical Nutrition, Medical University of Bialystok ul. Mieszka I 4B, 15-054 Bialystok, Poland; lucyna.ostrowska@umb.edu.pl (L.O.); joanna@zapolska.home.pl (J.L.); damian.pogodzinski@umb.edu.pl (D.P.); 2 Department of Biochemical Diagnostics, Medical University of Bialystok, ul. Waszyngtona 15A, 15-269 Bialystok, Poland; orywalk@umb.edu.pl; 3 Department of Statistics and Medical Informatics, Medical University of Bialystok, ul. Szpitalna 37, 15-295 Bialystok, Poland; urszula.cwalina@umb.edu.pl

**Keywords:** probiotics, cardiorespiratory fitness, body composition, long-distance running, inflammation

## Abstract

Use of probiotic supplements, the benefits of which have not been proven in sportspeople, is becoming more widespread among runners. The aim of this study was to evaluate the effect of a multi-strain probiotic on body composition, cardiorespiratory fitness and inflammation in the body. The randomised, double-blind study included 66 long-distance runners. The intervention factor was a multi-strain probiotic or placebo. At the initial and final stages of the study, evaluation of body composition and cardiorespiratory fitness was performed and the presence of inflammation determined. In the group of men using the probiotic, an increase in lean body mass (*p* = 0.019) and skeletal muscle mass (*p* = 0.022) was demonstrated, while in the group of women taking the probiotic, a decrease in the content of total body fat (*p* = 0.600) and visceral fat (*p* = 0.247) was observed. Maximum oxygen consumption (VO_2max_) increased in women (*p* = 0.140) and men (*p* = 0.017) using the probiotic. Concentration of tumour necrosis factor-alpha decreased in women (*p* = 0.003) and men (*p* = 0.001) using the probiotic and in women (*p* = 0.074) and men (*p* = 0.016) using the placebo. Probiotic therapy had a positive effect on selected parameters of body composition and cardiorespiratory fitness of study participants and showed a tendency to reduce inflammation.

## 1. Introduction

Long-distance running, which includes half marathons (approximate distance 21 km), marathons (approximately 42 km), ultramarathons (commonly around 100 km) or 24 h runs, belongs to a group of endurance sports [1]. Endurance running is characterised by long duration (from 30 min to several hours) and varying intensity, depending on the type of sporting competition and capabilities of competitors. It is usually performed at 30–60% of the maximum oxygen uptake (VO_2max_). These values may change depending on the stamina of runners, distance covered, type of terrain and environmental conditions. The popularity of endurance sports is increasing every year. Marathons and ultramarathons are no longer competitions for professionals—they are also open to amateur runners [2]. It is estimated that in 2015 more than 40,000,000 Americans ran regularly. In Denmark, 25% of the population run regularly, considering it the main physical activity [2]. In Poland, male (61%) inhabitants of large cities (>500,000 people) aged 25–34 years are more likely to run than women [3].

Individuals who practice endurance sports often seek to significantly reduce their body mass, particularly fat mass, in order to increase exercise capacity and improve speed, motor skills and overall body efficiency [4]. A carefully planned reduction in excess body fat may contribute not only to an improvement in speed but also enhanced dynamics and more effective use of oxygen by working muscles [5]. As demonstrated in a study by Manore [6] in 2012, athletes who trained with a weight attached to their waist equal to 5% of their own body weight experienced an almost 3% decrease in exercise capacity compared to training without a load. Training of runners should be based on improving muscular endurance without excessive muscle building. Greater skeletal muscle mass will improve athletes’ strength, endurance and performance [7]. It is worth noting that individuals training for a marathon or ultra-marathon frequently focus only on running and strengthening lower limbs, forgetting about general training and building skeletal muscle mass in the upper limbs and the trunk. Body composition analysis may indicate increased water content in this group of athletes, which may be related to enhanced skeletal muscle mass. It is also indicated that BMI is not sufficient to assess the nutritional status of athletes because of the fact that only body weight and height are considered in the evaluation. In physically active people, the use of BMI is not recommended [8].

Body composition may have an impact on athletes’ faster running times [9,10]. In endurance sports, particularly during sustained, low-intensity activity, it is mainly aerobic capacity that is activated. It depends on the amount of circulating blood, cardiac output or lung capacity and ventilation [11,12]. When defining the performance status of athletes, it is worth focusing on the maximum oxygen consumption by the body, known as VO_2max_. Improvement in VO_2max_ is influenced by high intensity training. In men aged 20–25 years, this parameter is equal to 45–55 mL × kg body weight^−1^ × minute^−1^. After the age of 20, this value decreases by approximately 1% per year [11,12]. Professional athletes display a VO_2max_ equal to 70 mL × kg body weight^−1^ × minute^−1^ [13,14]. Physical performance is influenced by properly planned training (taking into consideration its duration and intensity), genetic and psychological factors (motivation) and the external environment (temperature, altitude, ambient humidity). From a physiological viewpoint, physical performance is influenced by the capacity of the cardiovascular, respiratory, nervous and endocrine systems and metabolism of muscle cells. Balanced nutrition and appropriate dietary supplementation also play a crucial role in physical performance [13,14].

Despite its many health benefits, physical activity may have adverse effects. Depending on the intensity and duration of an endurance exercise, the risk of nausea, vomiting, abdominal pain and diarrhoea may increase [15]. Upper respiratory tract infections or chronic inflammation may also be observed. The risk of injury also increases [16]. Probiotic strains that modulate intestinal microbiota and the occurrence of gastrointestinal disorders or upper respiratory tract infections (URTIs) are continually sought [17]. An increase in the incidence of URTIs is observed during training periods, mainly in the autumn, winter and spring. The influence of probiotic strains on the performance of participants in various sports disciplines also seems interesting. Probiotic strains, their dosage and type of physical activity in which measurable health benefits could be observed have not been precisely defined to date [18]. The aim of this study was to evaluate the effect of a multi-strain probiotic on body composition, cardiorespiratory fitness and inflammation.

## 2. Materials and Methods

The randomised, double-blind study included 70 individuals who met the study inclusion criteria: males and females aged 20–60 years old who performed moderate or intense physical activity based on long-distance running. All subjects provided informed consent for study participation prior to study commencement. The study was conducted in accordance with the Declaration of Helsinki, and the study protocol was approved by the Bioethics Committee of the Medical University (No. RI-002/81/2017). The study was performed in the spring and autumn, i.e., the periods of most intense physical activity associated with sporting competitions entry.

**Figure 1:**
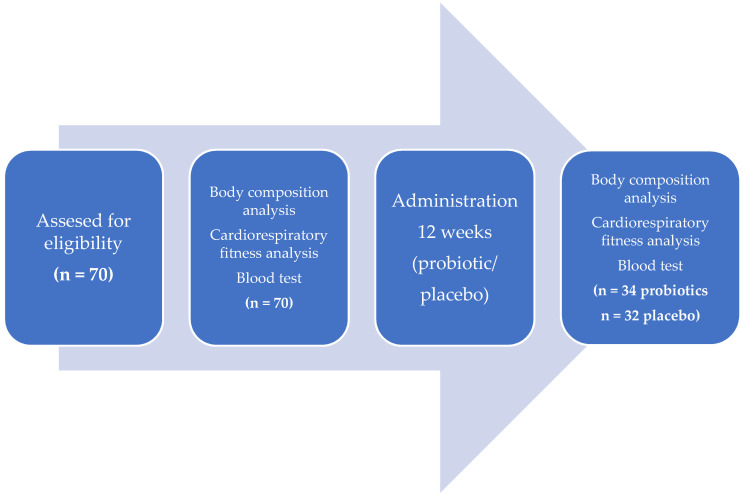
Experimental design: 70 subjects met the study inclusion criteria. Stage 1 of the study included body composition analysis, cardiorespiratory fitness analysis, and a blood test to assess for the presence of inflammation. For 12 weeks following stage 1, participants took either a probiotic (*n* = 35) or a placebo (*n* = 35). After this time, stage 2 of the study was conducted in which tests analogous to stage 1 were performed on 34 people from the probiotic group and 32 people from the placebo group.

In the study, a multi-strain probiotic SANPROBI BARRIER (Sanprobi Ltd. Szczecin, Poland) or a placebo were used. The probiotic, which is readily available at Polish pharmacies, contains the following bacterial strains: *Bifidobacterium lactis* W52, *Lactobacillus brevis* W63, *Lactobacillus casei* W56, *Lactococcus lactis* W19, *Lactococcus lactis* W58, *Lactobacillus acidophilus* W37, *Bifidobacterium bifidum* W23 and *Lactobacillus salivarius* W24 in a dose of 2.5 × 10^9^ CFU/g (1 capsule). Study participants ingested 2 capsules of the supplement twice a day (morning and evening) for a period of 3 months. Every second study participant was randomly selected for the probiotic group (*n* = 35), while the remainder constituted the placebo group (*n* = 35). The study was entirely anonymous and the subjects provided written informed consent for study participation. Sixty-six individuals participated in the second stage of the study. One participant from the probiotic group did not attend the final appointment, for which no reason was provided, while from the placebo group, two subjects did not participate in the final stage of the study due to the fact of an injury and one for an undisclosed reason. The study was performed in two stages as shown in Figure 1:

### 2.1. Body Composition

At the initial and final stages of the study, all competitors underwent body composition analysis using the InBody770 analyser (Seoul, South Korea). The body composition analyser uses bioelectrical impedance analysis to establish a person’s body composition. The following parameters were assessed: body weight (kg), body fat content in kilograms and percentages, visceral fat content (cm^2^), skeletal muscle mass (kg) and body water content (kg). Participants were informed about the need to fast for 8–12 h and the necessity to avoid physical activity for at least 24 h before the test. They were asked to refrain from drinking liquids for 1 h prior to the test. None of the participants had a pacemaker, which is an absolute exclusion criterion for bioimpedance analysis.

### 2.2. Cardiorespiratory Fitness Analysis

In order to determine the cardiorespiratory fitness of study participants, the following appliances were used: the Fitmate MED device (Cosmed, Rome, Italy), which comprehensively determines cardiovascular and respiratory capacity, and a medical treadmill adapted to this type of analysis by HpCosmos (Nussdorf—Traunstein, Germany). The Fitmate MED device is equipped with a sensor that enables the measurement of inhaled and exhaled air, and a mask containing disposable mouthpieces and antibacterial filters. To monitor heart rate, a Garmin sport tester, model HRM-Tri 010-10997-09 (Olathe, KS, USA), was used. In the present study, the Bruce protocol treadmill test was used. The test was divided into 5 stages of 3 min duration. At each stage, the gradient and speed of the treadmill were elevated to increase the work output. Stage 1 of the Bruce protocol was performed at 2.7 km/h and a 10% gradient; stage 2 was performed at 4 km/h and a 12% gradient; stage 3 was performed at 5.5 km/h mph and a 14% gradient; stage 4 was performed at 6.8 km/h and a 16% gradient; stage 5 was performed at the speed of 8 km/h and a 18% gradient.

Study participants were instructed that the cardiorespiratory fitness test should be conducted at least two weeks after intense physical activity (e.g., marathon, ultramarathon). During the test, the maximal test protocol was used in order to obtain more test parameters. The test was terminated when the participant achieved 90% HR_max_ or was unable to continue. All study participants completed the test, attaining 90% HR_max_.

### 2.3. Inflammation

Tests were performed after collecting 10 mL of venous blood (from the ulnar vein) from each study participant. Concentrations of C-reactive protein (CRP) and tumour necrosis factor (TNF)-alpha) were determined twice—at the initial and the final stages of the study. For the CRP test, blood was centrifuged and after the clot was separated in the blood serum, tests were performed. To determine TNF-alpha, blood was collected on a clot, centrifuged and after the clot was separated, the obtained serum was stored at –80 degrees Celsius until the tests were performed. The enzyme-linked immunosorbent assay (ELISA) method was used to determine the concentration of TNF-alpha. The R&D System kits (Bio-Techne, Minneapolis, MN, USA) and Anthos ELISA Reader (Salzburg, Austria) were utilised according to the manufacturer’s instructions.

### 2.4. Statistics

Statistical analysis of the obtained results was performed using the statistical program STATISTICA 13.3 by StatSoft. Descriptive statistics were prepared by designating mean values, standard deviations, ranges of minimum and maximum values and medians for quantitative features. Due to the size of the studied groups, normal distribution of the quantitative variables was not assessed. The analysis used non-parametric methods. Wilcoxon’s test was used to compare dependent samples. The Mann–Whitney U test was used to compare the study group and the control group. Statistical significance was set at *p* < 0.05.

## 3. Results

Selected parameters associated with body composition, cardiorespiratory fitness and levels of inflammation in study participants were analysed. The average age of the female subjects in the probiotic group was 37.21 ± 8.09 years, while the average age of the female subjects in the placebo group was 33.33 ± 8.73 years. The average age of the male subjects in the probiotic group was 40.85 ± 8.32 years, while in the placebo group it was 38.61 ± 8.84 years (differences were not statistically significant). Study participants ran approximately 40 km per week and the average number of hours spent training ranged from 4.6 to 8.4 h per week and did not differ significantly among the groups.

Body composition variables, broken down by gender, were compared prior to and following the intervention. After a three-month intervention, both groups of women showed an increase in total body water (TBW), lean body mass (LBM) and skeletal muscle mass (SMM), but the differences were not statistically significant. In the group of women taking the probiotic supplement, a decrease in body fat (in kilograms and percentages) and visceral fat (VAT) was observed, but the differences were not statistically significant. In the placebo group of women, a marginal decrease in the percentage of body fat was observed as well as a decrease in visceral fat. However, these differences were not statistically significant. The results are presented in Table 1.

Following a three-month intervention, a statistically significant increase in total body water (*p* = 0.019), an increase in lean body mass (*p* = 0.019) and an increase in skeletal muscle mass (*p* = 0.022) were found. No statistically significant differences were found in the placebo male group. The results are presented in Table 2.

Following a three-month intervention, an increase in all parameters of cardiovascular and respiratory efficiency, except for breathing reserve and anaerobic threshold (AT), was demonstrated in the probiotic group of women. However, the differences were not statistically significant. In the placebo group of women, a reduction in the VO_2max_, minute ventilation (Ve), exercise capacity, breathing reserve and anaerobic threshold was observed. The differences were not statistically significant. A statistically significant increase (*p* = 0.027) in respiratory frequency (Rf) was demonstrated in the group of placebo women. The results are presented in Table 3.

Following a three-month intervention, a statistically significant increase in maximum oxygen uptake VO_2max_ (*p* = 0.017), minute ventilation (Ve) (*p* = 0.013), functional capacity (FC) (*p* = 0.036), breathing reserve (*p* = 0.020) and exercise capacity (*p* = 0.036) was observed in the group of men taking the probiotic supplement. Such favourable changes were not observed in the control group. The results are presented in Table 4.

In order to determine the presence of inflammation, concentrations of C-reactive protein and TNF-alpha were analysed. The results are presented in Table 5. In the group of women using the probiotic supplement, the concentration of C-reactive protein decreased (2.17 ± 3.50 mg/L versus 0.87 ± 1.14 mg/dL). The differences were not statistically significant. In the placebo group of women, a marginal decrease in C-reactive protein concentration was observed but the differences were not statistically significant. In the group of men using the probiotic supplement, a decrease in the concentration of this parameter was also observed, but it was not as marked as in the group of women (1.75 ± 2.27 mg/L versus 1.63 ± 2.81 mg/L). The differences were not statistically significant. In the placebo group of men, a decrease in C-reactive protein concentration was demonstrated (1.40 ± 2.26 mg/L versus 1.09 ± 1.31 mg/L), but the differences were not statistically significant.

Following a three-month intervention, the concentration of TNF-alpha decreased in both the female and male groups. In the group of women taking the probiotic supplement, a statistically significant (*p* = 0.003) reduction in TNF-alpha concentration (10.82 ± 1.61 pq/mL versus 9.39 ± 1.43 pq/mL) was observed. However, a decrease in TNF-alpha concentration in the placebo group of women was close to statistical significance (*p* = 0.074). In the group of men taking the probiotic supplement, a statistically significant (*p* = 0.001) reduction in the concentration of this parameter (11.30 ± 0.85 pq/mL versus 9.68 ± 1.46 pq/mL) was observed. In the placebo group of men, a significant reduction in TNF-alpha was also demonstrated (*p* = 0.016).

## 4. Discussion

Physical activity is an element of a healthy lifestyle [19]. It is considered a preventative measure against many diseases including type 2 diabetes [20], diseases of the cardiovascular system [21], depression [22] and sarcopenia [23]. Effective physical training improves body composition parameters including an increase in skeletal muscle mass and a decrease in subcutaneous and visceral fat levels [24]. Body composition parameters, such as skeletal muscle mass or adipose tissue content, are very important when an athlete’s exercise capacity and speed attained in endurance sports are considered [9]. In the present study, increases in lean body mass and skeletal muscle mass were observed in the two groups of women studied. In the group of women taking the probiotic supplement, reductions in total fat content and body fat percentage were observed (differences were not statistically significant). On the other hand, in the group of women taking the placebo, an increase in total fat content was noted (differences were not statistically significant). In a study by Huang et al. (2019), a similar relationship was demonstrated. After six weeks of probiotic supplementation, a more significant decrease in body fat percentage was observed in the group using the supplement in comparison to the placebo group. Additionally, skeletal muscle mass increased significantly in the group of subjects using the probiotic compared to the placebo [24].

The final evaluation of male subjects from the probiotic group demonstrated a statistically significant increase in total water content (*p* = 0.019), lean body mass (*p* = 0.019) and skeletal muscle mass (*p* = 0.022) in comparison to the initial assessment. No statistically significant differences were found in the placebo group of men. The results differ from our research findings published in 2012. Hottenrott et al. [25], in their three-month study, observed a reduction in body fat percentage (22.5% versus 21%), visceral fat content (5.6 kg versus 4.7 kg) and a decrease in lean body mass (51.8 kg versus 50.8 kg). The results of the body compositions of elite runners presented in a study by Jang et al. [19] differed from those obtained in the present investigation. The above analysis demonstrated lower lean body mass (51.8 ± 4.1 kg), lower percentage of adipose tissue (9.2 ± 1.6%) and decreased total content of adipose tissue (5.5 ± 1.1 kg) in comparison to our study. The lower body fat content in professional athletes reported in the study may result from intense physical activity and duration of practicing the sport (average 7 years). Low adipose tissue content allows for achieving higher speeds when running and has a positive effect on the musculoskeletal system.

In the present study, probiotic intervention had a positive effect on the reduction of visceral fat content in female participants. In a study by Szulińska et al. [26], three-month supplementation with probiotic strains, such as the ones used in the present study (*Bifidobacterium bifidum* W23, *Bifidobacterium lactis* W51, *Bifidobacterium lactis* W52, *Lactobacillus acidophilus* W37, *Lactobacillus brevis* W63, *Lactobacillus casei* W56, *Lactobacillus salivarius* W24, *Lactococcus lactis* W19 and *Lactococcus lactis* W58), caused a statistically significant decrease in visceral adipose tissue mass (*p* = 0.033) compared to the placebo group. However, the study was performed on postmenopausal women. Low visceral fat content is crucial to reducing the risk of cardiovascular disease [27]. Furthermore, results similar to those obtained in the present investigation were reported in a study in which the probiotic strain *Lactobacillus gasseri* BNR17 was administered [28]. Twelve-week probiotic supplementation resulted in a statistically significant (*p* = 0.038) reduction in visceral fat content compared to the placebo group.

In the present study, an increase in all parameters of cardiorespiratory fitness, except for breathing reserve and anaerobic threshold, was demonstrated in the group of women using the probiotic supplement. By contrast, in the group of women taking the placebo, a reduction in maximum oxygen uptake, minute ventilation, functional and exercise capacity was revealed. In the group of men taking the probiotic supplement, a statistically significant increase in maximum oxygen uptake (VO_2max_) (38.22 ± 5.99 mL/kg/min versus 41.05 ± 8.02 mL/kg/min; *p* = 0.017), minute ventilation (Ve) (*p* = 0.013), functional capacity (*p* = 0.036), breathing reserve (*p* = 0.020) and exercise capacity (*p* = 0.036) was observed compared to men taking the placebo. The present investigation demonstrated that maximum oxygen uptake values attained by runners were significantly lower than those reported in a study by Huang et al. The authors showed that in the placebo group, the average VO_2max_ was 60.1 ± 2.2 mL/kg/min, while in the group using probiotics it was 61.8 ± 1.5 mL/kg/min. The study, similar to the present investigation, indicated that probiotic therapy has a positive impact on the effectiveness of training which, in turn, improves parameters of cardiorespiratory fitness in physically active people [24].

Delaying fatigue allows one to exercise more effectively and for longer. Furthermore, a “healthy microbiome” can improve the absorption and use of carbohydrates and other key nutrients, resulting in longer and more effective training (use of ingested carbohydrates and “protection” of muscle and liver glycogen). It also facilitates body regeneration [29]. Probiotic therapy, by positively influencing the composition of the intestinal microbiota, may reduce fatigue in individuals engaging in sports. This was confirmed in a study by Shing et al. [30] that demonstrated that four-week supplementation with a multi-strain probiotic extended the average duration of physical activity (running) in a hot environment to the time extreme fatigue was felt. As shown in other studies, the probiotic strain *L. plantarum* PS128 can significantly increase the concentration of branched amino acids in plasma (even by 24–69% compared to the initial concentration) and improve the efficiency of physical exercise, which was demonstrated by comparing individuals participating in sports to the placebo group (*p* = 0.05). The authors of the study concluded that the probiotic strain *L. plantarum* PS128 may be a potential, ergogenic “aid” to increasing the intensity of physical training and improving physiological adaptation to exercise [31]. Another study from 2018 showed that the probiotic strain *Lactobacillus plantarum* TWK10 leads to an improvement in endurance capacity, possibly through various regulatory mechanisms. After six weeks of supplementation, athletes demonstrated a significant improvement in exercise performance. The conclusions of the study are consistent with those of the previous study by the same authors in which the exercise performance of athletes using *Lactobacillus plantarum* TWK10 supplementation increased by 36.76% compared to the placebo group [24]. The authors of the study report, however, that further analyses are needed to confirm the effectiveness and mechanism of probiotic supplementation. This was also confirmed by a meta-analysis published in 2019 [32]. It reported only six scientific studies evaluating the effect of probiotic strains on the cardiorespiratory fitness, two of which revealed a beneficial effect (one of them was conducted on mice). There is a lack of literature on the effect of probiotic strains on athletes’ performance and most of the published studies concern animal models.

The occurrence of gastrointestinal disorders in athletes may be caused by chronic inflammation resulting from considerable physical exertion as well as chronic dysbiosis [30]. Following a three-month probiotic/placebo intervention, the present study revealed a decrease in the serum concentration of C-reactive protein both in male and female subjects. As demonstrated in a meta-analysis from 2016 encompassing 83 randomized and non-randomized trials, physical activity may reduce the concentration of C-reactive protein. The decrease may be explained by an improvement in the body composition of individuals playing sports (i.e., lower body weight and percentage of body fat) [33]. However, in another study from 2015, a one-time, 60 min physical activity resulted in an increase in serum C-reactive protein levels (observed only in women compared to men). The study was conducted in a group of footballers, not marathon runners, but the nature of work output allows for comparison of the concentration of this parameter in both groups. An increase in CRP concentration may be explained by the involvement of various mechanisms in the regulation of the acute phase under various conditions. Therefore, assessment of this index before and immediately after a physical activity may be a valuable tool for evaluating the metabolic reaction during an aerobic exercise [34].

In the present study, inflammation was also assessed using serum concentrations of TNF-alpha. It was demonstrated that serum concentrations of TNF-alpha decreased in all studied groups, and the differences showed high statistical significance (in the group of probiotic women: *p* = 0.003; probiotic men: *p* = 0.001; placebo men: *p* = 0.016). A decrease in TNF-alpha serum concentration in the group of women taking the placebo was close to statistical significance (*p* = 0.074). The results of a study by Lamprecht et al. in which athletes took a probiotic or a placebo for 14 weeks demonstrated that all subjects had higher than the norm TNF-alpha concentration, which is contradictory to the results obtained in the present study. Moreover, after 14 weeks of supplementation, the concentration of TNF-alpha decreased in the group using the probiotic compared to those taking the placebo. However, the results were not statistically significant [35]. Similar results were obtained in a study by West et al. [17] in which the concentration of TNF-alpha decreased in the group of people using probiotics compared to the placebo group. Huang et al. demonstrated that a group of 16 triathletes had higher TNF-alpha serum concentration values at the beginning of the study in comparison to the present study. Following a three-week intervention with *L. plantarum* PS128 or a placebo, a more marked increase in TNF-alpha concentration was observed in the placebo group immediately after physical activity compared to the group using the probiotic in which the concentration also increased, although the increase was marginal. Differences between the results showed statistical significance (*p* = 0.016) [31].

Reducing inflammation in the body is important for preventing and decreasing the incidence of gastrointestinal disorders. Reducing the occurrence of these conditions or eliminating them entirely allows for more effective training, which translates into improved preparation for sporting competitions and better results. The intestinal barrier is also crucial for the absorption and use of the main nutrients [36]. Strenuous exercise, which causes inflammation of intestinal cells, increases the phosphorylation of tight-junction proteins and, consequently, enhances their permeability. This, in turn, leads to increased susceptibility to infections and gastrointestinal problems [35]. The literature reports that intensive training results in the excessive production of IL-6 and TNF-alpha [37].

Differences between male and female subjects may be due to the fact of various reasons. They may result from the type and intensity of physical activity undertaken by the participants, which may impact on body composition [24]. In addition, the diet of runners should be considered, which may affect the content of adipose tissue and other body composition parameters. Gastrointestinal disorders are also a problem for runners [38]. It is likely that the decreased occurrence of these conditions has an impact on the effectiveness of training and, thus, improves parameters of body composition and cardiorespiratory fitness.

## 5. Conclusions

A three-month multi-strain probiotic intervention had a positive effect on the parameters of body composition of study participants (a statistically significant increase in lean body mass and skeletal muscle mass in men and a statistically insignificant decrease in the content of total and visceral adipose tissue in women).

The beneficial effect of probiotic therapy on the parameters of cardiorespiratory fitness in individuals playing endurance sports (statistically significant in men and statistically insignificant in women) was demonstrated.

The assessment of the parameters of inflammation in runners showed no significant differences between the probiotic and placebo groups.

## Figures and Tables

**Table 1:** Comparison of body composition variables of women from the study and control groups at the initial and final stages of intervention.

	Probiotic Women (*n* = 14)					Placebo Women (*n* = 6)					
	Initial Stage (P)		Final Stage (F)			Initial Stage (P)		Final Stage (F)			
Parameter	Mean ± SD	Median	Mean ± SD	Median	*p* *	Mean ± SD	Median	Mean ± SD	Median	*p* *	*p* **
TBW (kg)	34.82 ± 3.30	34.25	35.24 ± 3.58	35.10	0.504	36.18 ± 2.02	35.90	37.03 ± 2.00	36.65	0.248	0.535
LBM (kg)	47.61 ± 4.50	46.90	48.18 ± 4.92	48.10	0.600	49.48 ± 2.76	48.95	50.71 ± 2.71	50.25	0.172	0.482
BFM (kg)	15.00 ± 3.98	14.80	14.57 ± 4.34	13.80	0.600	17.01 ± 8.12	16.40	17.11 ± 9.35	17.10	0.916	0.836
PBF (%)	23.82 ± 5.12	24.25	23.28 ± 5.59	23.05	0.916	24.65 ± 8.59	25.60	24.18 ± 10.26	26.10	0.600	0.772
VAT (cm^2^)	62.13 ± 21.04	60.50	59.37 ± 22.62	56.05	0.247	61.33 ± 27.20	70.00	61.18 ± 26.20	70.70	0.833	0.679
SMM (kg)	26.29 ± 2.72	25.90	26.57 ± 2.92	26.35	0.779	27.35 ± 1.61	26.85	28.10 ± 1.73	27.85	0.248	0.322

* Statistical significance determined between the initial and final stages. ** Statistical significance determined between the probiotic versus placebo groups at the final stage. Total body water, (TBW); lean body mass (LBM); body fat mass, (BFM); percent of body fat, (PBF); visceral fat, (VAT); skeletal muscle mass, (SMM).

**Table 2:** Comparison of body composition variables of men from the study and control groups at the initial and final stages of intervention.

	Probiotic Men (*n* = 20)					Placebo Men (*n* = 26)					
	Initial Stage (P)		Final Stage (F)			Initial Stage (P)		Final Stage (F)			
Parameter	Mean ± SD	Median	Mean ± SD	Median	*p* *	Mean ± SD	Median	Mean ± SD	Median	*p* *	*p* **
TBW (kg)	47.43 ± 3.56	47.80	48.14 ± 3.82	48.60	0.019	49.20 ± 6.05	50.70	49.65 ± 6.00	50.55	0.061	0.327
FFM (kg)	64.73 ± 4.81	65.20	67.74 ± 5.23	66.40	0.019	67.15 ± 8.33	69.35	67.79 ± 8.29	69.00	0.063	0.338
BFM (kg)	14.62 ± 4.88	14.55	15.39 ± 4.64	14.60	0.184	13.83 ± 5.00	12.40	13.56 ± 4.88	13.15	0.431	0.067
PBF (%)	18.17 ± 5.05	17.95	18.76 ± 4.67	18.40	0.286	16.86 ± 4.86	15.65	16.54 ± 5.13	16.15	0.242	0.118
VAT (cm^2^)	61.80 ± 22.25	61.00	63.14 ±2 1.51	61.50	0.460	59.05 ± 21.33	59.05	58.06 ± 20.82	57.50	0.594	0.306
SMM (kg)	36.65 ± 2.86	37.35	37.29 ± 3.10	38.00	0.022	38.07 ± 4.98	39.45	38.46 ± 4.95	39.15	0.056	0.212

* Statistical significance determined between the initial and final stages. ** Statistical significance determined between the probiotic versus placebo groups at the final stage.

**Table 3:** Analysis of selected parameters of cardiorespiratory fitness of women from the probiotic and placebo groups at the initial and final stages of intervention.

	Probiotic Women (*n* = 14)					Placebo Women (*n* = 6)					
	Initial Stage (P)		Final Stage (F)			Initial Stage (P)		Final Stage (F)			
Parameter	Mean ± SD	Median	Mean ± SD	Median	*p* *	Mean ± SD	Median	Mean ± SD	Median	*p* *	*p* **
VO_2max_ (mL/kg/min)	34.02 ± 5.30	32.60	35.90 ± 6.16	35.25	0.140	36.98 ± 11.34	31.95	36.06 ± 8.85	33.00	0.600	0.302
Ve (L/min)	57.24 ± 10.54	56.00	61.24 ± 14.2	58.15	0.271	63.51 ± 15.25	60.15	63.48 ± 9.92	62.05	0.753	0.433
Rf (1/min)	30.25 ± 7.50	30.70	31.91 ± 8.57	32.05	0.300	30.06 ± 4.05	29.85	33.48 ± 4.02	34.15	0.027	0.386
HR (bpm)	150.28 ± 10.38	152.00	155.50 ± 9.1	158.00	0.208	153.50 ± 12.14	156.00	157.83 ± 13.16	161.00	0.093	0.967
FeO_2_ (%)	16.24 ± 0.47	16.19	16.25 ± 0.34	16.21	0.875	16.12 ± 0.36	16.12	16.17 ± 0.47	16.26	0.600	0.836
FC (METS)	9.72 ± 1.52	9.35	10.25 ± 1.7	10.05	0.151	10.56 ± 3.25	9.15	10.30 ± 2.53	9.40	0.600	0.248
Max VO_2_/HR (mL/beat)	14.10 ± 2.08	13.95	14.46 ± 2.47	13.98	0.432	15.63 ± 2.06	15.15	15.03 ± 2.25	14.70	0.401	0.247
Breathing reserve (%)	53.42 ± 8.95	56.00	50.50 ± 12.63	51.50	0.363	51.16 ± 9.32	53.00	50.66 ± 5.64	52.00	0.600	0.804
Exercise capacity (mL/kg/min)	34.02 ± 5.30	32.60	35.90 ± 6.16	35.25	0.934	36.98 ± 11.34	31.95	36.06 ± 8.85	33.00	0.710	0.302
AT (mL/kg/min)	19.97 ± 4.49	19.75	19.27 ± 3.02	18.65	0.868	21.40 ± 6.59	19.00	20.08 ± 6.81	18.05	0.772	0.868

* Statistical significance determined between the initial and final stages. ** Statistical significance determined between the probiotic versus placebo group at the final stage. Minute ventilation, (Ve); respiratory frequency, (Rf); heart rate, (HR); functional capacity (---), (FC(METS)); anaerobic threshold, (AT).

**Table 4:** Analysis of selected parameters of cardiorespiratory fitness of men from the probiotic and placebo groups at the initial and final stages of intervention.

	Probiotic Men (*n* = 20)					Placebo Men (*n* = 26)					
	Initial Stage (P)		Final Stage (F)			Initial Stage (P)		Final Stage (F)			
Parameter	Mean ± SD	Median	Mean± SD	Median	*p* *	Mean ± SD	Median	Mean ± SD	Median	*p* *	*p* **
VO_2max_ (mL/kg/min)	38.22 ± 5.99	37.15	41.05 ± 8.02	41.10	0.017	42.34 ± 706	42.65	43.86 ± 7.58	43.40	0.286	0.313
Ve (L/min)	79.78 ± 17.11	79.65	87.78 ± 20.52	84.90	0.013	93.50 ± 23.54	92.15	97.94 ± 28.85	92.95	0.258	0.253
Rf (1/min)	30.37 ± 5.87	30.25	31.61 ± 6.62	32.10	0.232	34.30 ± 9.15	35.95	33.92 ± 9.18	36.85	0.892	0.277
HR (bpm)	150.20 ± 7.77	151.00	152.10 ± 7.68	151.00	0.156	152.23 ± 7.45	152.00	155.19 ± 9.55	155.00	0.119	0.876
FeO_2_ (%)	16.10 ± 0.49	16.19	16.18 ± 0.45	16.18	0.121	16.21 ± 0.66	16.31	16.21 ± 0.78	16.30	0.706	0.363
FC (METS)	10.91 ± 1.70	10.60	11.67 ± 2.32	11.70	0.036	12.10 ± 1.98	12.15	12.56 ± 2.20	12.40	0.170	0.340
Max VO_2_/HR (mL/beat)	20.21 ± 3.49	20.30	21.27 ± 4.29	22.15	0.102	22.51 ± 3.67	22.30	23.08 ± 3.92	23.70	0.492	0.287
Breathing reserve (%)	50.70 ± 10.13	50.00	45.75 ± 12.63	47.50	0.020	45.07 ± 15.41	51.00	45.26 ± 13.75	48.00	0.558	0.128
Exercise capacity (mL/kg/min)	38.21 ± 6.01	37.15	40.86 ± 8.14	41.10	0.036	42.42 ± 7.18	42.65	44.07 ± 7.87	43.40	0.196	0.363
AT (mL/kg/min)	21.05 ± 4.21	20.40	20.35 ± 3.33	20.75	0.346	21.85 ± 4.06	21.55	20.13 ± 3.58	20.45	0.850	0.340

* Statistical significance determined between the initial and final stages. ** Statistical significance determined between the probiotic versus placebo groups at the final stage.

**Table 5:** Alterations in C-reactive protein (CRP) and tumour necrosis factor (TNF)-alpha concentrations in men and women from the probiotic and placebo groups between the initial and final stages of intervention.

	**Probiotic Women** **(*n* = 14)**					**Placebo Women** **(*n* = 6)**					
	**Initial Stage** **(P)**		**Final Stage (F)**			**Initial Stage** **(P)**		**Final Stage (F)**			
**Parameter**	**Mean ± SD**	**Median**	**Mean ± SD**	**Median**	*p* *	**Mean ± SD**	**Median**	**Mean ± SD**	**Median**	*p* *	*p* **
CRP (mg/L)	2.17 ± 3.50	0.85	0.87 ± 1.14	0.50	0.151	1.81 ± 2.17	0.95	1.21 ± 1.09	0.85	0.916	0.433
TNF-alpha (pg/mL)	10.82 ± 1.61	10.50	9.39 ± 1.43	9.48	0.003	10.57 ± 1.45	11.27	8.85 ± 1.21	8.36	0.074	0.693
**Probiotics Men** **(*n* = 20)**						**Placebo Men** **(*n* = 26)**					
	**Initial Stage** **(P)**		**Final Stage (F)**			**Initial Stage** **(P)**		**Final Stage (F)**			
**CRP (mg/L)**	1.75 ± 2.27	0.85	1.63 ± 2.81	1.00	0.459	1.40 ± 2.26	0.70	1.09 ± 1.31	0.70	0.330	0.334
**TNF-alpha (pg/mL)**	11.30 ± 0.85	11.21	9.68 ± 1.46	9.43	0.001	10.66 ± 0.95	10.71	9.78 ±1.69	8.87	0.016	0.327

* Statistical significance determined between the initial and final stages. ** Statistical significance determined between the probiotic versus placebo group at the final stages.

## Trial Registration

ClinicalTrials.gov Identifier: NCT04530929.
